# The concept of a cementless isoelastic monoblock cup made of highly cross-linked polyethylene infused with vitamin E: radiological analyses of migration and wear using EBRA and clinical outcomes at mid-term follow-up

**DOI:** 10.1186/s12891-021-03981-8

**Published:** 2021-01-23

**Authors:** Yama Afghanyar, Sebastian Joser, Jonas Tecle, Philipp Drees, Jens Dargel, Philipp Rehbein, Karl Philipp Kutzner

**Affiliations:** 1 Department of Orthopaedic Surgery, St. Josefs Hospital Wiesbaden, Beethovenstr. 20, 65189 Wiesbaden, Germany; 2 Department of Orthopaedics and Traumatology, University Medical Centre of the Johannes Gutenberg-University of Mainz, Langenbeckstraße 1, 55131 Mainz, Germany

**Keywords:** Total hip arthroplasty, Cementless acetabular cup, Vitamys, EBRA, Migration, Wear

## Abstract

**Background:**

The newest generation of cementless titanium-coated, isoelastic monoblock cup with vitamin E-blended highly cross-linked polyethylene (HXLPE) was introduced to the market in 2009. The aim of the present study was to obtain mid-term follow-up data including migration and wear analyses.

**Methods:**

This prospective study investigated 101 primary total hip arthroplasty (THA) cases in 96 patients treated at a single institution. Patients were allowed full weight-bearing on the first day postoperatively. Harris hip score (HHS) and pain and satisfication on a visual analogue scale (VAS) were assessed at a mean follow-up of 79.0 months. Migration and wear were assessed using Einzel-Bild-Roentgen-Analyse (EBRA) software. Radiological acetabular bone alterations and complications were documented.

**Results:**

At mid-term follow-up (mean 79.0 months, range: 51.8–101.7), 81 cases with complete clinical and radiological data were analyzed. Utilisable EBRA measurements were obtained for 42 hips. The mean HHS was 91.1 (range 38.0–100.0), VAS satisfaction was 9.6 (range 6.0–10.0), VAS rest pain was 0.2 (range 0.0–4.0), and VAS load pain was 0.6 (range 0.0–9.0). Mean migration was 0.86 mm (range: 0.0–2.56) at 24 months and 1.34 mm (range: 0.09–3.14) at 5 years, and the mean annual migration rate was 0.22 (range: − 0.24–1.34). The mean total wear was 0.4 mm (range: 0.03–1.0), corresponding to a mean annual wear rate of 0.06 mm per year (range: 0.0–0.17). Radiographic analysis did not reveal any cases of osteolysis, and no revision surgeries had to be performed.

**Conclusions:**

After using vitamin-E blended HXLPE in cementless isoelastic monoblock cups, there were no obvious signs of osteolysis or aseptic loosening occurred. No patients required revision surgery after mid-term follow-up. Cup migration and wear values were well below the benchmarks considered predictive for potential future failure.

**Trial registration:**

The trial registration number on ClinicalTrials.gov: NCT04322916 (retrospectively registered at 26.03.2020).

**Supplementary Information:**

The online version contains supplementary material available at 10.1186/s12891-021-03981-8.

## Background

Total hip arthroplasty (THA) is one of the most common operations performed worldwide [1]. The most challenging requirement for THA is long implant survival and aseptic component loosening is one of the most prevalent indications for revision surgery [2, 3]. In recent decades, substantial efforts have been made to avoid or prolong aseptic loosening and mechanical implant failure, but these issues will remain majors challenge in the future [4–7].

**Fig. 1:**
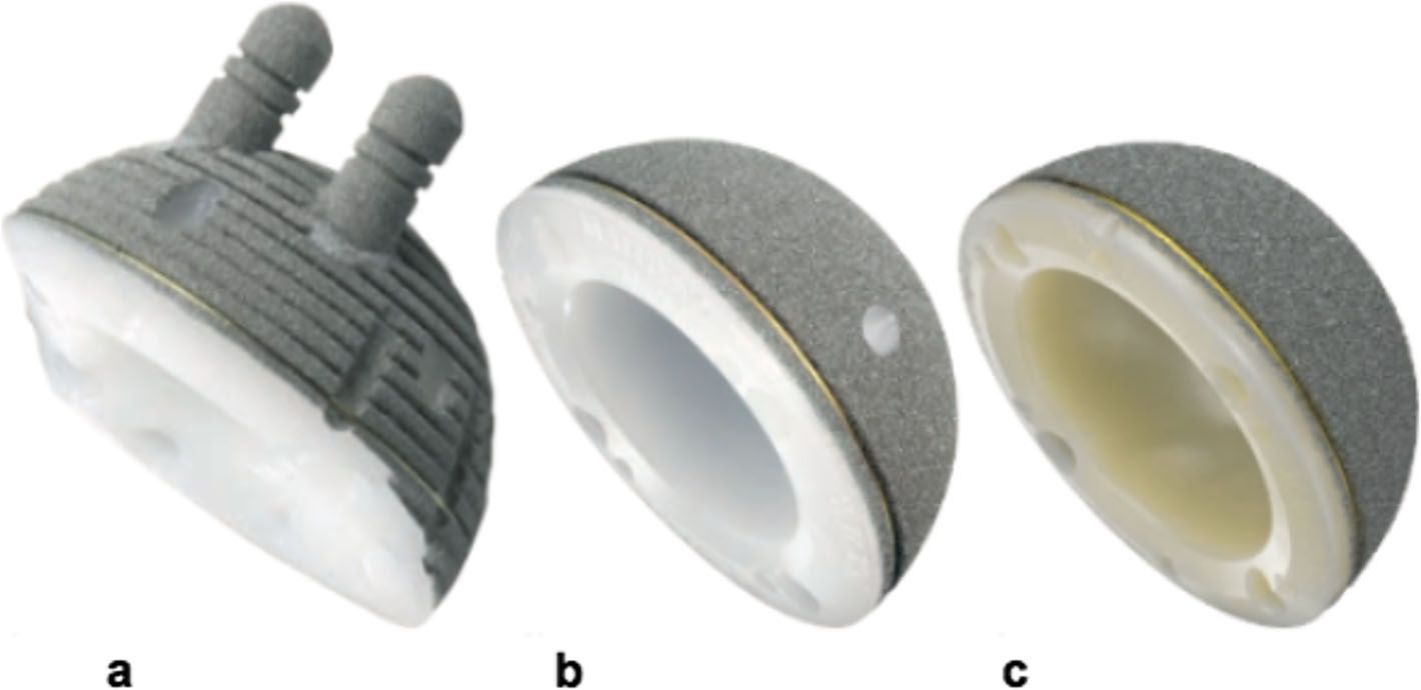
**a** RM Classic cup; **b** RM Pressfit cup; **c** RM Pressfit vitamys cup. (Mathys Ltd., Bettlach, Switzerland)

The concept of a cementless monoblock cup was first introduced in 1983 [4, 8] (Fig. 1a). It was made of ultra-high-molecular-weight polyethylene (UHMWPE) coated with titanium for primary bone fixation, and two pegs provided rotational stability [9]. A 20-year follow-up revealed a survival rate of 94.4% using aseptic loosening as the endpoint [4].

The second-generation monoblock cup was introduced in 2002 (Fig. 1b). It was also made of UHMWPE, but both pegs were removed. The longevities of both generations of monoblock cups were limited by wear and oxidative degeneration of the UHMWPE [4, 10].

The third generation was introduced to the market in 2009 (Fig. 1c). To improve the properties, it used a new generation of highly cross-linked polyethylene that is protected by the antioxidant vitamin E [11–13]. This type of polythylene promised significantly lower wear rates [2, 11–14]. Although some studies have shown that vitamin E-stabilized highly-crosslinked UHMW-PE (HXLPE) has similar wear properties to HXLPE without vitamin E, it does have improved fatigue strength and is protected from oxidative destruction [11, 15–18].

Osteolysis is induced by polyethylene wear and potentially causes loosening and implant failure. Dumbleton et al. defined a threshold of 0.1 mm per year, below which osteolysis is rarely observed [19]. Early cup migration is another major indicator for late aseptic loosening. Several studies used “Einzel-Bild-Roentgen-Analyse” (EBRA) [20] measurements and identified implant migration > 2 mm during the first 2 years as an established risk factor for implant failure by interfering osteointegration [6, 21–25].

The primary aim of this study was to analyse the radiological outcomes of the newest generation of monoblock vitamin E-blended HXLPE cup in a mid-term follow-up. The migration pattern and wear were assessed to identify potentially undesireable results at an early stage, and clinical outcomes were obtained.

## Methods

The present prospective observational study investigated 101 primary THA cases in 96 patients treated at a single institution between March 2010 and September 2011. All procedures performed were in accordance with the 1964 Helsinki Declaration, and institutional ethical approval was obtained (FF 154/2017). All patients gave their verbal and written permission to participate prior to inclusion. The results are reported according to the STrengthening the Reporting of OBservational studies in Epidemiology (STROBE) Statement.

The inclusion criteria were age between 20 and 85 years and a candidate for primary THA. The demographics of all enrolled patients are presented in Table 1.

**Table 1:** Patient demographics

	Total included Mean (range)	Total with clinical and radiological FU at mid-term Mean (range)
Number of hips, n	101	81
Age, years	69.4 (50.7–84.3)	68.0 (50.7–84.0)
BMI, kg/m^2^	27.5 (19.3–41.5)	27.7 (20.6–41.5)
Diagnosis, n (%)		
Primary osteoarthrosis	94 (93.1%)	76 (93.8%)
Secondary osteoarthrosis	2 (2.0%)	1 (1.2%)
Femoral head necrosis	3 (3.0%)	3 (3.7%)
Femoral neck fracture	1 (1.0%)	0 (0.0%)
Congenital dysplasia	1 (1.0%)	1 (1.2%)

*BMI* body mass index, *FU* follow-up

The RM Pressfit vitamys cup (Mathys Ltd., Bettlach, Switzerland) (Fig. 1c) was used for all THAs. The body of the cup is made of HXLPE stabilized with vitamin E [5, 11, 18]. The titanium coating was developed to maintain the natural elastic properties of isoelastic polyethylene and promote secondary stability [8, 11, 18]. Primary stability is achieved by equatorial pressfit, and up to four screws can be inserted into predefined holes in case of insufficient acetabular coverage or in soft or sclerotic bone. The femoral components and head components used are listed as supplementary data.

In all cases, a minimally invasive, antero-lateral approach using a standardized surgical technique was employed [26]. Surgery was performed by experienced consultant surgeons, and the mean duration was 56.4 min (range: 30.0–93.0). All patients started physiotherapy and were allowed full weight-bearing ambulation on the first postoperative day.

The clinical and radiological follow-up included a maximum of six timepoints: preoperative, 6 weeks, 6 months, 12 months, 2 years, and 5 years. Perioperative complications and adverse events were documented. Clinical examination was performed using the Harris Hip Score (HHS) and rest pain and load pain on a visual analogue scale (VAS) before surgery and during follow-up.

**Fig. 2:**
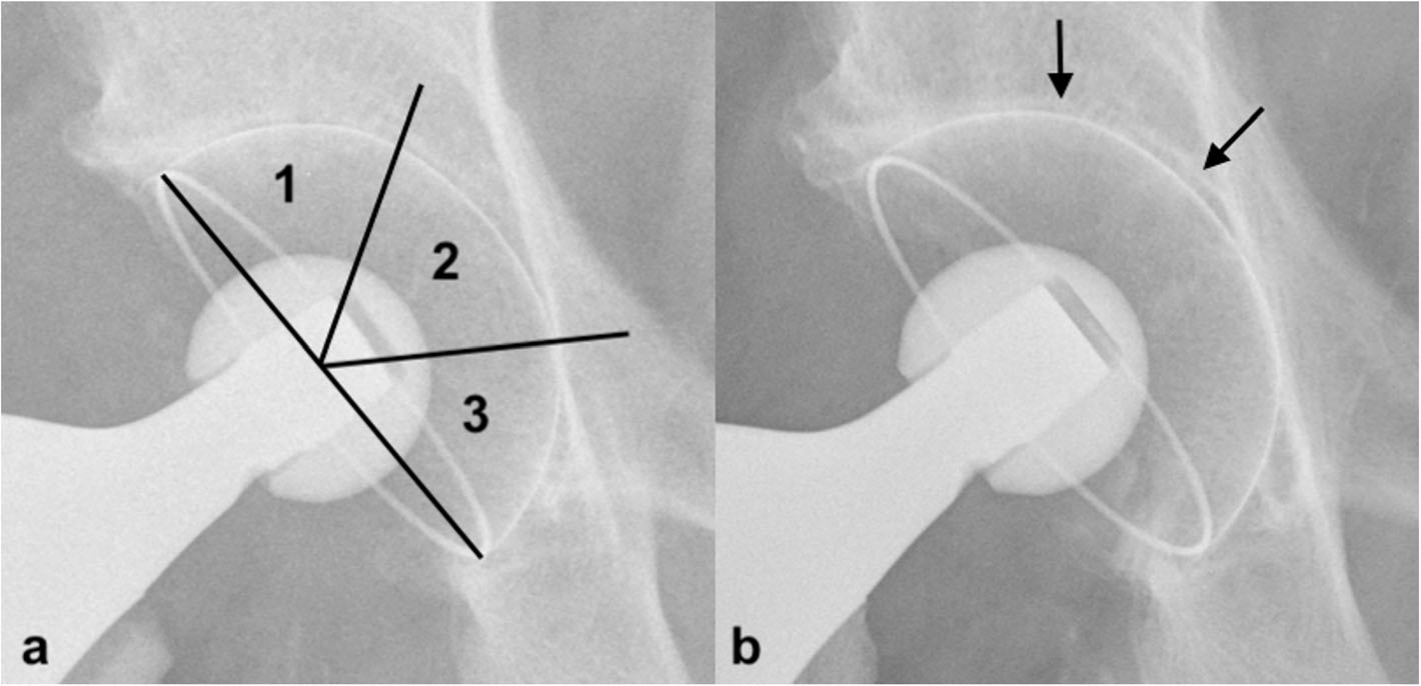
Radiographs of an RM Pressfit vitamys cup (Mathys Ltd. Bettlach, Switzerland) immediately after surgery (**a**) and at 5-year follow-up (**b**). The patient showed signs of a sclerotic line in Zones 1 and 2

The radiological evaluation of osseointegration and migration of the cup was based on standardized anterior-posterior pelvic radiographs. Lucent lines and osteolysis were analyzed according to Engh et al. [27] and defined in the zones described by DeLee and Charnley [28] (Fig. 2a). Heterotopic ossifications were documented according to Brooker [29].

At mid-term follow-up, a retrospective evaluation of the radiographs using EBRA was performed by one observer to analyse the migration pattern and detect potential wear of the polyethylene. The methology was originally reported by Krismer et al. [20]. Briefly, migration is being investigated in two orientations: horizontally (x-axis = cupx) and vertically (y-axis = cupy). Decreasing and increasing values in the horizontal direction imply medial and lateral migration, respectively. Correspondingly, increasing values in the y-direction refer to migration in the cranial or proximal direction, while decreasing y-values signify distal movement. However, given that biplanar analyses are not possible, the anteroposterior motion is discounted by the EBRA method. The accuracy of EBRA has been validated within 1 mm [21]. Since radiographs are only accepted by the EBRA software if all reference lines can be accurately located, there is a high failed evaluation rate. Loosening was defined as total migration increase of 0.5 mm per year [30, 31]. Total cup migration was claculated as the vector summation of x and y-axis migration according to Wyatt et al. [5].

Creep and wear rate were also calculated using pelvic radiographs and EBRA software. A frame drawn from tangents to prominent structures defined the position of the pelvis. From the digitized points, the software calculated the best-fit circle for the femoral head or the best fitting ellipse for the contrast wire and the distances from each digitized coordinate of the implant [32]. The displacement of the head center was calculated relative to the cup center in the frontal plane (transverse and longitudinal axis), and the wear rate for each subgroup was calculated for each time interval between radiographic examinations [32]. Displacement of the head center during the first postoperative year is considered a combination of creep and wear, while penetration of the femoral head after the first year may be defined as actual wear [2, 33].

### Statistical analysis

All statistical analyses were carried using standard descriptive statistics such as mean ± standard deviation (SD) and median (i.e., 50% percentile) and range. For all outcomes related to migration and wear, 2-sided 95% confidence intervals and 25 and 75% percentiles are provided as supplementary measures of variation. The aim of the study was to assess migration and wear outcomes with sufficient precision, so no explicit statistical tests were carried out. Qualitative categorical values are shown as number and percentage. All statistical analyses were performed using SAS software 9.4 (SAS Institute, Cary, North Carolina, USA).

## Results

**Fig. 3:**
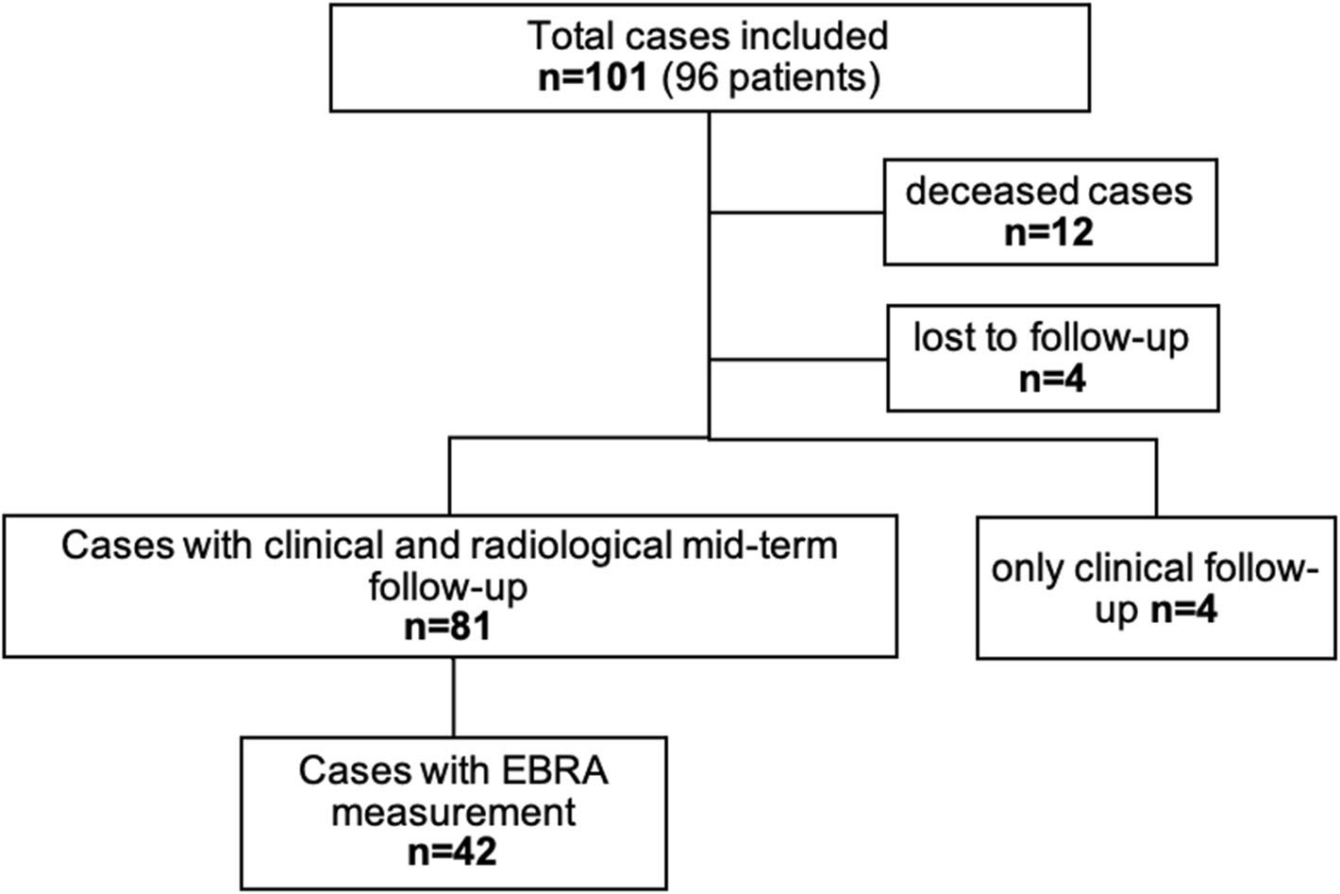
Study flow diagram

After 5 years, 12 patients were deceased with the investigated implants in situ. All deaths were unrelated to the surgical procedure and did not occur within the first postoperative year. Four cases were lost to follow-up. Only clinical follow-up could be obtained for another four symptom-free patients who decline additional radiography. Thus, 81 cases with complete clinical and radiological data could be analyzed (Fig. 3). The mean follow-up time was 79.0 months (range: 51.8–101.7). None of the patients required cup-related revision due to aseptic loosening, mechanical failure, or any other reason after 5 years.

Two (2%) fissures of the femur were observed intraoperatively, but both healed uneventfully after cerclage wiring. The other 99 (98%) patients did not exeprience intraoperative complications. During the early postoperative period, there were four hematomas, one case of femoral nerve palsy, and one superficial infection that was treated successfully by antibiotic therapy. During follow-up, two patients developed periprosthetic fractures of the femoral component due to trauma, without any involvement of the acetabular component. No cup-specific complications were observed.

The mean range of motion increased from a preoperative value of 92° flexion (range: 50–120) to 114° (range: 85–130). The clinical outcomes are shown in Table 2.

**Table 2:** Longitudinal clinical outcomes

FU	Mean	SD	Median	95% CI		Percentiles		Range	
				Min	Max	25%	75%	Min	Max
VAS rest pain (pts)									
Pre-op	5.0	3.3	5.0	4.4	5.7	2.0	8.0	0.0	10.0
6–12 weeks	1.1	1.0	1.0	0.6	1.5	0.0	2.0	0.0	3.0
6 months	0.2	0.3	0.1	−0.1	0.6	0.0	0.4	0.0	0.7
12 months	0.5	1.2	0.0	0.3	0.8	0.0	1.0	0.0	8.0
24 months	0.1	0.3	0.0	0.0	0.2	0.0	0.0	0.0	2.0
5 years	0.2	0.7	0.0	0.0	0.3	0.0	0.0	0.0	4.0
VAS load pain (pts)									
Pre-op	7.7	2.2	8.0	7.2	8.1	6.7	9.8	1.0	10.0
6–12 weeks	1.5	1.4	1.0	0.8	2.2	0.3	2.2	0.0	4.0
6 months	0.6	0.6	0.8	−0.1	1.4	0.0	1.0	0.0	1.4
12 months	1.1	1.7	0.0	0.7	1.4	0.0	2.0	0.0	8.0
24 months	0.3	0.5	0.0	0.2	0.4	0.0	0.8	0.0	2.0
5 years	0.6	1.7	0.0	0.3	1.0	0.0	0.0	0.0	9.0
VAS satisfaction (pts)									
Pre-op	1.7	2.0	1.0	1.4	2.1	0.0	3.0	0.0	8.0
6–12 weeks	8.8	0.8	9.0	8.5	9.2	8.5	9.1	7.3	10.0
6 months	9.5	0.5	9.4	8.9	10.1	9.2	10.0	9.0	10.0
12 months	9.2	1.4	10.0	8.9	9.5	8.8	10.0	2.0	10.0
24 months	9.7	0.5	10.0	9.6	9.8	9.5	10.0	8.0	10.0
5 years	9.6	0.9	10.0	9.4	9.8	10.0	10.0	6.0	10.0
Harris Hip Score (pts)									
Pre-op	49.9	15.0	52.0	46.9	52.9	41.5	60.5	9.0	95.0
6–12 weeks	83.1	10.4	85.0	78.2	88.0	75.5	91.5	64.0	99.0
6 months	91.8	4.0	91.0	86.9	96.7	91.0	95.0	86.0	96.0
12 months	91.1	11.6	96.0	88.5	93.7	88.0	99.0	45.0	100.0
24 months	95.2	5.6	97.0	94.0	96.4	93.0	99.0	75.0	100.0
5 years	94.0	9.9	97.0	91.9	96.2	93.0	100.0	38.0	100.0
Flexion (°)									
Pre-op	92.4	12.1	90.0	90.0	94.7	90.0	100.0	50.0	120.0
6–12 weeks	101.0	13.8	100.0	94.5	107.5	90.0	110.0	65.0	130.0
6 months	109.0	13.9	115.0	91.8	126.2	110.0	115.0	85.0	120.0
12 months	116.3	12.0	120.0	113.6	118.9	110.0	125.0	85.0	130.0
24 months	120.2	9.4	120.0	118.2	122.3	115.0	130.0	95.0	130.0
5 years	113.7	10.9	115.0	111.3	116.1	110.0	120.0	85.0	130.0

*CI* confidence interval, *FU* follow-up, *VAS* visual analogue score

Before surgery, the mean HHS was 49.9 points (range: 9.0–95.0). At mid-term follow-up, the mean HHS was 94.0 points (range: 38.0–100).

The mean rest pain on VAS decreased from 5.0 (range: 0–10) preoperatively to 0.2 at mid-term, and mean load pain on VAS decreased from 7.7 (range: 1.0–10.0) to 0.6 (range: 0.7–9.0). Satisfaction on VAS increased from 1.7 (range: 0.0–8.0) to 9.5 (range: 0.9–10.0) after 5 years.

The mid-term radiographic results are presented in Table 3. One patient showed a lucent line in zone 2 and partly in zone 1 (Fig. 2b). None showed evidence of osteolysis of the acetabulum after 5 years. Heterotopic bone formation was observed in 8.6% (7) of patients. Six (7.4%) showed Brooker I, and one (1.2%) patient showed Brooker II.

**Table 3:** Longitudinal radiological outcomes

	5-Year follow-up	
	Frequency (n)	Percent (%)
Inclination		
< 30°	0	0.0
30–34°	1	1.2
35–39°	2	2.5
40–44°	7	8.6
45–49°	28	34.6
50–54°	36	44.4
> 54°	7	8.6
Lucent lines		
No	81	100.0
Yes	0	0.0
Osteolysis		
No	81	100.0
Yes	0	0.0
Heterotopic ossification		
No	74	91.4
Yes	7	8.6
Brooker I	6	7.4
Brooker II-III	1	1.2
Brooker IV	0	0.0

The mean number of radiographs available for EBRA evaluation was 3.88 (range 3.0–5.0) per patient, and data from 42 patients contributed to the EBRA analysis at mid-term follow-up.

Mean cup migration at mid-term follow-up was 1.34 mm (range: 0.09–3.14). The migration rate per year decreased from 0.36 mm per year (range: − 3.55–4.02) at 12 months to 0.22 mm per year (range: − 0.24–1.34) at 5 years (Table 4).

**Table 4:** EBRA measurements of total migration and total wear at each follow-up

	Months	n	Mean	SD	Median	Min	Max
**Total migration (mm)**	0	44	0.00	0.00	0.00	0.00	0.00
	3	36	0.34	0.41	0.23	0.00	2.07
	12	50	0.80	0.71	0.56	0.00	2.81
	24	48	0.86	0.62	0.70	0.00	2.56
	60	42	1.34	0.63	1.26	0.09	3.14
**Total migration rate (mm/year)**	3	36	1.89	2.57	0.88	0.00	11.53
	12	50	0.72	0.63	0.48	0.00	2.35
	24	48	0.29	0.21	0.21	0.00	0.83
	60	42	0.21	0.10	0.19	0.01	0.47
**Total wear (mm)**	0	44	0.00	0.00	0.00	0.00	0.00
	3	36	0.11	0.12	0.06	0.00	0.54
	12	50	0.18	0.17	0.15	0.00	0.78
	24	48	0.28	0.17	0.26	0.00	0.68
	60	42	0.40	0.24	0.32	0.03	1.00
**Total wear rate (mm/year)**	3	36	0.61	0.70	0.38	0.00	2.72
	12	50	0.17	0.15	0.13	0.00	0.67
	24	48	0.09	0.05	0.09	0.00	0.26
	60	42	0.06	0.04	0.05	0.00	0.17

*EBRA* Einzel-Bild-Roentgen-Analyse

During the first postoperative year, the mean combination of creep and wear resulted in 0.17 mm (range: 0.00–0.67). Mean total wear at mid-term follow-up was 0.40 mm (range: 0.03–1.00), and mean annual wear rate was 0.06 mm (range: 0.00–0.17) per year (Table 4). When considering the combination of creep and wear during the first year, the actual annual wear rate was 0.05 mm (range: − 0.06–0.21).

## Discussion

The aim of the present study was to assess mid-term radiological outcomes in THA and investigate the migration and wear rate of the third generation of a cementless isoelastic monoblock cup with vitamin E-blended HXLPE. At mid-term follow-up, almost no cup-related complications were observed, and none of the investigated implants required revision surgery. Radiologically, no direct signs of cup loosening were obvious, and no cases osteolysis were observed. Clinical outcomes were good, with very high patient satisfaction scores.

Early migration is considered a predictor of aseptic loosening [5, 34], and mean cup migration > 2 mm in the first 2 years was shown to correlate significantly with long-term aseptic loosening [21–25, 31]. The present investigation found a mean total migration of 1.34 mm at 5 years. Mean migration of 0.86 mm was seen at 2 years, which was far below the above-mentioned 2 mm limit. Additionally, loosening was defined as an overall migration increase of 0.5 mm per year [30, 31]. We measured a migration rate of 0.22 mm per year at 5 years, which was also far below the threshold for aseptic loosening.

Few previous publications have studied the newest cup generation. Wyatt et al. [5] also analyzed cup migration, however, but only a few EBRA measurements (*n* = 13) were included. They found a mean migration of 1.5 mm at 5 years, which was confirmed by the present study. Since most migration occurred within the first 12 weeks after surgery, the authors concluded it was due to the initial cup seating and all components can be considered stable thereafter [5]. The present study also found that migration stagnated at 1–2 years postoperatively, and secondary stabilization subsequently occurred in most cases. After the first 2 years and the onset of secondary stability, only slight further migration was observed.

The cause of migrations can be both inadequate initial fixation with insufficient primary stability or loss of fixation during follow-up [31, 34]. Both scenarios might indicate an increased risk of failure. However, minor migration over years is often asymptomatic [6, 34]. In the present study, the clinical results were excellent with no signs of failure despite minor continuous migration. Longer-term follow-up will be needed to confirm these findings.

Wyss et al. [6] investigated the second generation of the isoelastic monoblock cup (RM Pressfit, Mathys Ltd.) in a mid-term follow-up. Similar results to the present study were found, even though all surgeries were performed using a transgluteal approach, patients were only alowed partial weight bearing, and flexion was initially limited to 70° [6]. It is obvious that migration and subsequent loosening might reflect the quality of operative technique - particularly the reaming process - and implant selection [31]. One study reported a correlation between cup inclination and cup diameter with early migration [22]. However, the postoperative treatment protocol is likely to affect early migration. In the present study, all surgeries were performed using the minimallyinvasive anterolateral approach, theoretically making cup positioning more challenging and potentially affecting migration. It is remarkable that similar results were achieved in the present study compared to the investigation by Wyss et al. [6], although our postoperative treatment protocol was far more aggressive.

Polyeythelene wear is another indicator of aseptic loosening of endoprosthetic components and causes osteolysis of the acetabular and femoral bone stock. This aspect was considered in the present investigation. It is critical to further improve UHMWPE to decrease wear and potentially increase the lifetime of acetabular components.

Previous in vitro studies demonstrated that vitamin E-blended HXLPE improves fatigue strength and protects against oxidative damage [11, 15, 17, 18]. The protection of HXLPE with vitamin E could imbue excellent oxidation resistance [11, 13, 15, 35] and potentially reduce wear. Beck et al. performed mechanical in vitro testing and found a significantly lower wear rate of vitamin E-blended HXLPE compared to standard gamma-sterilized UHMWPE [11]. Decreasing wear rate and oxidative degeneration may reduce osteolysis, which in turn could decrease rates of aseptic loosening and failure.

An early study found that osteolysis was rarely observed in THA patients with wear below the threshold of 0.1 mm per year [19]. In the present investigation, annual wear rates were far below this benchmark at 0.06 mm per year. Since most femoral head displacement in the first postoperative year may be associated with creep, annual rates of actual wear are to be expected even lower. Dumbleton et al. found that below a rate of 0.05 mm per year, osteolysis will practically not occur [19]. Earlier studies using the second generation of the isoelastic monoblock cup reported slightly higher annual wear rates compared to the present results. Wyss et al. [6] and Lafon et al. [7] found 0.09 mm and 0.07 mm per year, respectively. Rochcongar et al. recently performed a prospective randomized controlled study comparing the RM Pressfit cup (UHMWPE) to the RM Pressfit vitamys cup (HXLPE/VitE) and reported that wear rates over the first 3 years following surgery were lower for HXLPE/VitE group [2]. This could directly affect the need for late revision surgery. These results were again confirmed in a recent randomized-controlled trial comparing annual wear rates of the RM Pressfit cup (UHMWPE) to those of the RM Pressfit vitamys cup (HXLPE/VitE). At 2 years [36] and 6 years [37], the wear rates were significantly lower for the HXLPE/VitE cup.

At mid-term follow-up, no adverse events occurred and none of the investigated cups showed signs of failure, and not a single revision surgery was necessary in our study. A sclerotic line in zone 2 was observed in some of the patients at the cup dome, without increasing the risk of subsequent loosening. This is likely because the aspheric design of the cup has a flattened dome. Osteointegration seemed to be complete and stable in zones 1 and 3 in those cases.

Moreover, our encouraging clinical results with marked improvements in functionality and activity level confirm earlier studies [2, 4–7, 9, 10, 14, 18] and strongly support the concept of a cementless isoelastic monoblock cup with vitamin-E blended HXLPE.

Some limitations of the present study have to be acknowledged. The first is the mid-term follow-up period of 5 years. Although only long-term results should be considered regarding implant survival, early evaluation of radiological alterations, migration, and wear is helpful to identify future undisirable results. Early migration analysis using EBRA has been established as a reference to long-term survival [5, 6, 34]. Second, the method used to measure migration and wear has lower accuracy than radiostereometric analysis (RSA) [32]. As the accuracy has been validated within 1 mm, our results should be interpreted with caution. Nevertheless, EBRA has become a widely used scientific method without the need to implant markers intraoperatively and thus reduces cost and effort. Measured wear might be greater with EBRA than when using RSA, for example, due to probable plastic cup deformation affecting contrast wire shape [21, 32]. Another important limitation is that the EBRA software failed to evaluate all available radiographs. The image requirements for EBRA measurement are considerable, leading to a high rate of radiographs being rejected by the EBRA software. In the present study, reliable EBRA measurements were only obtained in 42 out of 81 hips at mid-term. Since several radiographs were taken at different time points during the follow-up period, the mean migration and wear results are reliable.

## Conclusion

In the present study using vitamin-E blended HXLPE in cementless isoelastic monoblock cups, no signs of osteolysis were obvious and no cases of aseptic loosening occurred. There was no need for revision surgery at mid-term. Values for cup migration and wear stayed well below the benchmarks considered predictive for future failure. Long-term data will be needed to confirm these findings.

## Supplementary Information

**Additional file 1: Supplementary Data.** Femoral components and head components used

## Data Availability

The dataset generated and/or analyzed during the current study are not publicly available due to the high volume of data but are available from the corresponding author on reasonable request.

## Abbreviations

EBRA: Einzel-Bild-Roentgen-Analyse
HHS: Harris hip score
HXLPE: Highly cross-linked polyethylene
RSA: Radiostereometric analysis
THA: Total hip arthroplasty
UHMWPE: Ultra-high-molecular-weight polyethylene
VAS: Visual analogue scale
