# Supplementary material for: The concept of a cementless isoelastic monoblock cup made of highly cross-linked polyethylene infused with vitamin E: radiological analyses of migration and wear using EBRA and clinical outcomes at mid-term follow-up
Source: BMC Musculoskelet Disord. 2021 Jan 23;22:107. doi: 10.1186/s12891-021-03981-8 (PMC7827971; doi:10.1186/s12891-021-03981-8)
Supplement: Supplementary file 1 — Additional file 1: Supplementary Data. Femoral components and head components used [file 12891_2021_3981_MOESM1_ESM.docx]

**Supplementary Data**

Femoral components and head components used

| Femoral components | % | Head components | % |
| --- | --- | --- | --- |
| Nanos (Smith&Nephew) | 33.7 | Biolox (Smith&Nephew) | 38.7 |
| Marathon (Smith&Nephew) | 3.0 |  |  |
| ProxyPlus (Smith&Nephew) | 2.0 |  |  |
| twinSys (Mathys) | 27.7 | Bionit (Mathys) | 45.5 |
| optimys (Mathys) | 17.8 |  |  |
| Metha (B.Braun/Aesculap) | 12.8 | Biolox (B.Braun/Aesculap) | 12.8 |
| Mayo (Zimmer/Biomet) | 3.0 | Biolox Delta (Zimmer/Biomet) | 3.0 |
